# A modified and enhanced test setup for biomechanical investigations of the hindfoot, for example in tibiotalocalcaneal arthrodesis

**DOI:** 10.1186/s12891-016-1177-6

**Published:** 2016-07-29

**Authors:** Julia Evers, Martin Schulze, Dominic Gehweiler, Martin Lakemeier, Michael J. Raschke, Dirk Wähnert, Sabine Ochman

**Affiliations:** Department of Trauma-, Hand- and Reconstructive Surgery, University Hospital Muenster, Albert-Schweitzer-Campus 1, Building W1, 48149 Muenster, Germany

**Keywords:** Tibiotalocalcaneal arthrodesis, Hindfoot, Biomechanics, Intramedullary nailing

## Abstract

**Background:**

Tibiotalocalcaneal arthrodesis (TTCA) using intramedullary nails is a salvage procedure for many diseases in the ankle and subtalar joint. Despite “newly described intramedullary nails” with specific anatomical shapes there still remain major complications regarding this procedure. The following study presents a modified biomechanical test setup for investigations of the hindfoot.

**Methods:**

Nine fresh-frozen specimens from below the human knee were anaysed using the Hindfoot Arthrodesis Nail (Synthes) instrument. Quasi-static biomechanical testing was performed for internal/external rotation, varus/valgus and dorsal/plantar flexion using a modified established setup (physiological load entrance point, sledge at lever arm to apply pure moments). Additionally, a 3D optical measurement system was added to allow determination of interbony movements.

**Results:**

The mean *torsional* range of motion (ROM) calculated from the actuator data of a material testing machine was 10.12° (SD 0.6) compared to 10° (SD 2.83) as measured with the Optotrak® system (between tibia and calcaneus). The Optotrak showed 40 % more rotation in the talocrural joint.

Mean *varus/valgus* ROM from the material testing flexion machine was seen to be 5.65° (SD 1.84) in comparison to 2.82° (SD 0.46) measured with the Optotrak. The subtalar joint showed a 70 % higher movement when compared to the talocrural joint.

Mean ROM in the *flexion* test was 5.3° (SD 1.45) for the material testing machine and 2.1° (SD 0.39) for the Optotrak. The movement in the talocrural joint was 3 times higher compared to the subtalar joint.

**Conclusion:**

The modified test setup presented here for the hindfoot allows a physiological biomechanical loading. Moreover, a detailed characterisation of the bone-implant constructs is possible.

## Background

Tibiotalocalcaneal arthrodesis (TTCA) using an intramedullary nail is a salvage procedure for many diseases involving the talocrural and subtalar joint. Since the first report of arthrodesis with retrograde femoral nails, new generations of intramedullary nails have been designed in regard to the specific anatomy of the ankle.

**Fig. 1:**
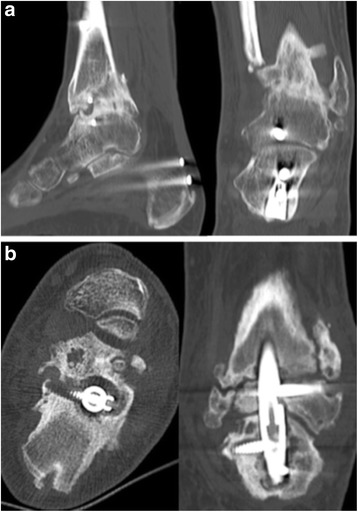
**a** Non-union of the subtalar joint after tibiotalocalcaneal arthrodesis by intramedullary nail with successful arthrodesis of the ankle joint. (68 year old male patient, 18 months after arthrodesis due to osteoarthritis and infection after pilon-fracture **b**) Complete failure with nail loosening and non-union of the ankle joint and the subtalar joint. (58 old male patient, 18 months after tibiotalocalcaneal arthrodesis due to varus osteoarthritis of the ankle and pes cavovarus correction)

Modifications, especially those regarding the development of a lateral bend in the distal part of the intramedullary nail, led to more stability and therefore less non-union rates because of the nail’s course, going through the calcaneus instead of the sustentaculum tali. In addition, with the reconstruction of the physiological hindfoot valgus, the simulation of a more conventional gait pattern was possible [1, 2]. In their biomechanical study, Mann et al. showed the importance of the course of the calcaneal screw from posterior to anterior [3]. These results could be confirmed by Means et al. [4]. But still there are major complications; especially the non-union of the upper ankle joint and/or subtalar joint are major problems, which follow this procedure (Fig. 1) [5–8].

It is common sense, that instability, especially torsional and shear stress, has negative impact on bone healing. In case of the hindfoot arthrodesis three bones and two joints are involved, which can cause instability und thus promote a non-union. Therefore, comparing overall construct stability is insufficient, attentions has to be directed to the stability of the talocrural and subtalar joint separately.

Actual biomechanical setups are able to compare the complete bone-implant- constructs only, without offering any differentiation of talocrural and subtalar joint movement. An established biomechanical method [9–11] was therefore modified and enhanced to address this disadvantage. Furthermore, the load entry point on the construct was adapted to mimic more physiological conditions. Additionally, a 3D motion tracking system was added to evaluate movements between all joint sections (tibia, talus, calcaneus) individually.

## Methods

### Specimens

Nine fresh-frozen human specimens from below the knee taken from our Institute of Anatomy were used for this study (five female, four male donors). Median age of the donors was 85.3 years (range: 77–95 years), four right and five left tibiae were available. The specimens were stored at -18C and thawed at room temperature for 24 h before testing.

For screening of pre-existing bony pathologies and implant size estimation, conventional radiographs in two planes (antero-posterior and lateral) were obtained. Additional bone mineral density of the cancellous bone in the calcaneus was measured by quantitative computed tomography (Somatom Definition, Siemens, Erlangen, Germany).

The specimens were prepared by removing all soft-tissue except for the stabilizing ligaments at the ankle such as the distal syndesmotic complex, the interosseous membrane, collateral ligaments and the capsule of the ankle and subtalar joints. The joint surfaces of the ankle and subtalar joints were left intact following former biomechanical studies [9, 12]. The forefoot was amputated at the Chopart’s joint. In contrast to the study of Klos et al. the fibula was not resected, in order to prevent additional instability [9]. Tibia and fibula were transected 30 cm above the ankle joint.

### Implant

**Fig. 2:**
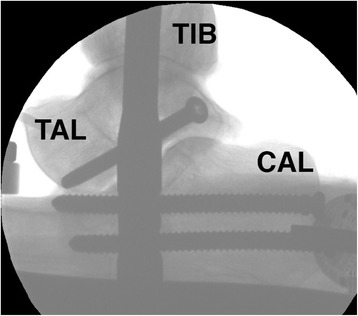
Lateral x-ray of a specimen before testing showing the Hindfoot Arthrodesis Nail (HAN) with two calcaneal, one talar locking screws for distal nail locking

For this study we used the Hindfoot Arthrodesis Nail (HAN, Depuy Synthes, West Chester, PA, USA) made of titanium alloy (Ti6Al7Nb). The HAN-nail shows a valgus bend of 12° distally. Nail locking was performed using two screws in the calcaneus, one screw in the talus and two screws in the tibia (Fig. 2). The nail used in this study had a length of 240 mm and a diameter of 10 mm (following x-ray measurement).

### Implantation

An experienced orthopaedic surgeon, following the manufacturers’ guidelines and using the adequate tools under c-arm control, performed implantation.

In contrast to the intraoperative procedure, the joint cartilage was not removed to ensure a standardized procedure.

### Biomechanical testing

The test setup used in this study was based on the publications of Mückley et al. and Klos et al. [9, 10]. They used an upside down positioning of the specimen with force application via a 80 mm lever arm using a connecting rod for varus/valgus and flexion testing, for torsional testing force was applied directly to the embedding [9].

**Fig. 3:**
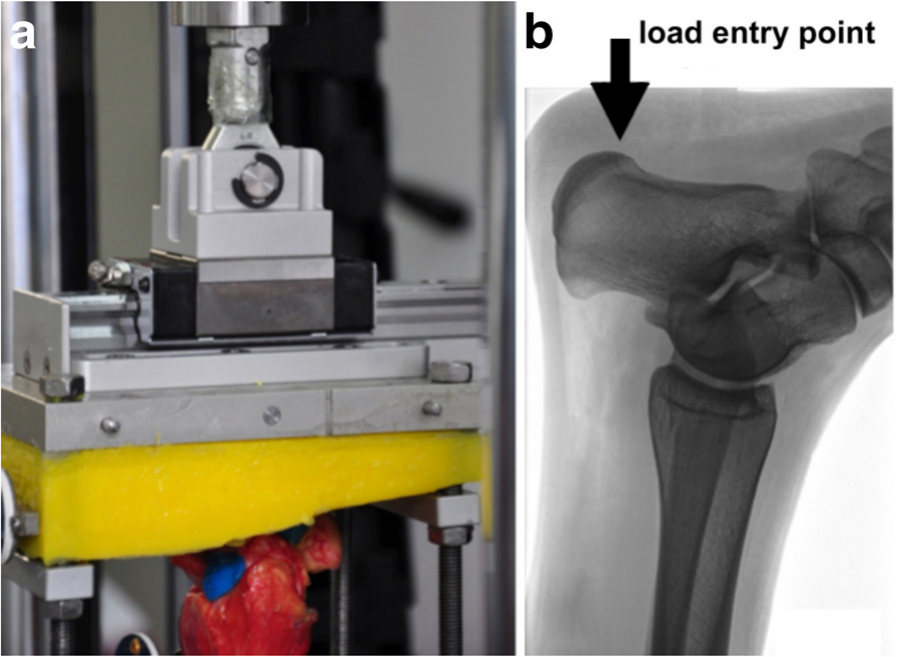
**a** Modification of the load application by adding a ball-bearing rail guide (sledge) at the suspension to exclude shear stress and to apply loads with a constant lever arm; **b** Shift of the load entry point to the tuber of the calcaneus to simulate physiological conditions. In contrast Klos et al. [7, 8] applied the load in extension of the tibia shaft axis

For mechanical testing a material testing machine (Instron 8874, Instron, High Wycombe, Bucks, United Kingdom) was used. We modified the test setup in two main aspects:

First, we included a ball-bearing rail guide (sledge) with negligible friction to the new lever arm instead of the connecting rod described by Mückley et al. and Klos et al. [11, 12]. The sledge was connected to the testing machine actuator with a type of cardanic suspension, which allowed small rotational movements in all directions. This refined suspension ensured a constant length of the lever arm of 80 mm in every position during varus/valgus and dorsi-/plantarflexion. In these motion planes, the sledge minimized shear stress on the specimen and allowed a more anatomical and especially standardized loading condition (Fig. 3a). The length of the lever arm was chosen following the work of Klos et al. [9, 12]. Embedding the tuber calcanei on a marked position (at the base plate) and using a cross-laser for the alignment of the specimens on the machine table ensured standardization of the lever arm length to 80 mm. Secondly, the load entry point was moved to the tuber of the calcaneus (from the anterior processes of the calcaneus as described by Mückley et al. and Klos et al.) [9, 11, 12] to simulate a physiologic loading of the construct, according to a normal heel strike during the gait pattern (Fig. 3b) [13–15]. A physiologic load entry point is from special interest for torsional loading and the comparison of intramedullary implants. Due to the varying nail entry point torsional stability depends on the relation of nail and load entry point. An especially unstable situation can be imagined, when nail and load entry point are identical.

Before embedding, all screw holes and the subtalar joint were covered using modelling clay to prevent any influence on the biomechanical properties, especially artificial fusion of the subtalar joint due to misplaced bone cement and infiltration of the cement into the medullary canal. First the calcaneus was embedded using Technovit (Technovit 3040 Heraeus Kulzer GmbH, Wernheim, Germany) and a special casing to allow a radiological view on the calcaneus. Afterwards, the tibia and fibula were embedded, verifying the adjustment and positioning by a cross-line laser. To exclude shear stress, the proximal embedding was performed on the testing machine.

**Fig. 4:**
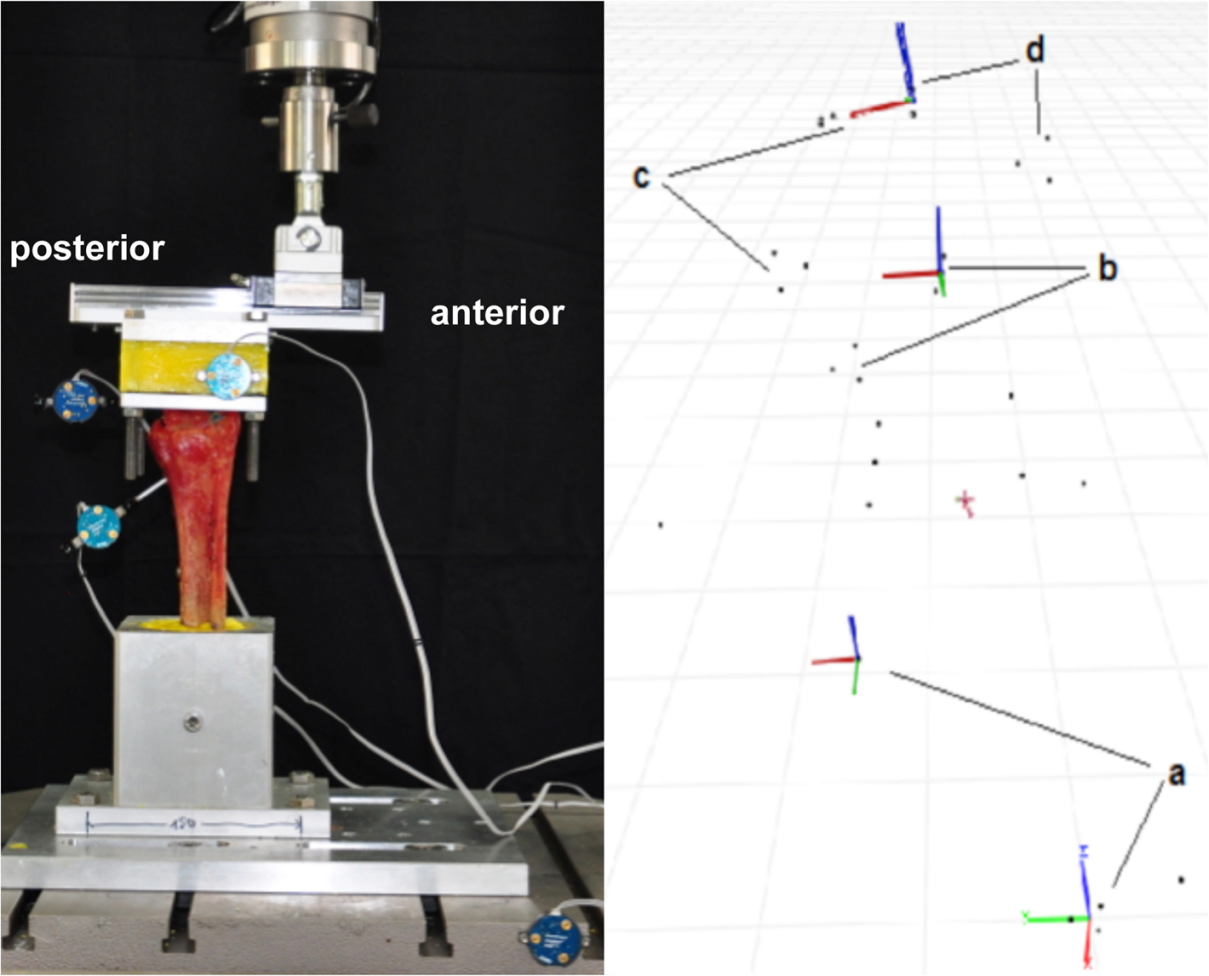
Test setup for dorsi-/plantarflexion (*left*) with markers on machine table, tibia, talus and calcaneus embedding. First Principles display (*right*) showing x-axis in *red*, y-axis in green and z-axis in *blue*. **a** global coordinate system and coordinate system of the Instron machine table; **b** tibia; **c** talus; **d** calcaneus. The *black dots* represent reference points to create the coordinate systems

Finally, a 3D optical motion measurement system was attached to the specimens (Fig. 4; Optotrak Certus, NDI Europe GmbH, Radolfzell, Germany). Four active marker triplets were used: one at the tibial shaft, one at the talus, one at the embedding of the calcaneus and one at the machine table (global reference coordinate system). The NDI software (First Principles Version 2, NDI Europe GmbH, Radolfzell, Germany) allowed calculation of all relative movements within the standardized coordinate systems. Data were recorded using a frequency of 25 Hz. The accuracy of the NDI measurement of translational motions was within ±0.03 mm and had a resolution of 0.01 mm; rotational accuracy was 0.0757 ± 0.121° [16].

All biomechanical tests were performed in load-control mode, machine data (time, cycle, angle, moment, axial displacement, force) were recorded at a rate of 64 Hz. The forces and resulting moments were selected due to former publications [9–11, 17]. Biomechanical testing was performed according to the following protocol:

- quasi-static tests (20 cycles) in

- o dorsi-/plantarflexion (±62.5 N equal to ±5 Nm) at 0.5 Hz
- o varus/valgus (±62.5 N equal to ±5 Nm) at 0.5 Hz
- o torsion in external-/internal rotation (±5 Nm) at 1°/sec

### Analysis

After 10 cycles of loading (to exclude setting behaviour) 10 cycles of mechanical loading have been applied to the specimens. For these 10 cycles the range of motion and neutral zone has been determined for every specimen, for further evaluation a mean value has been calculated for every specimen.

**Fig. 5:**
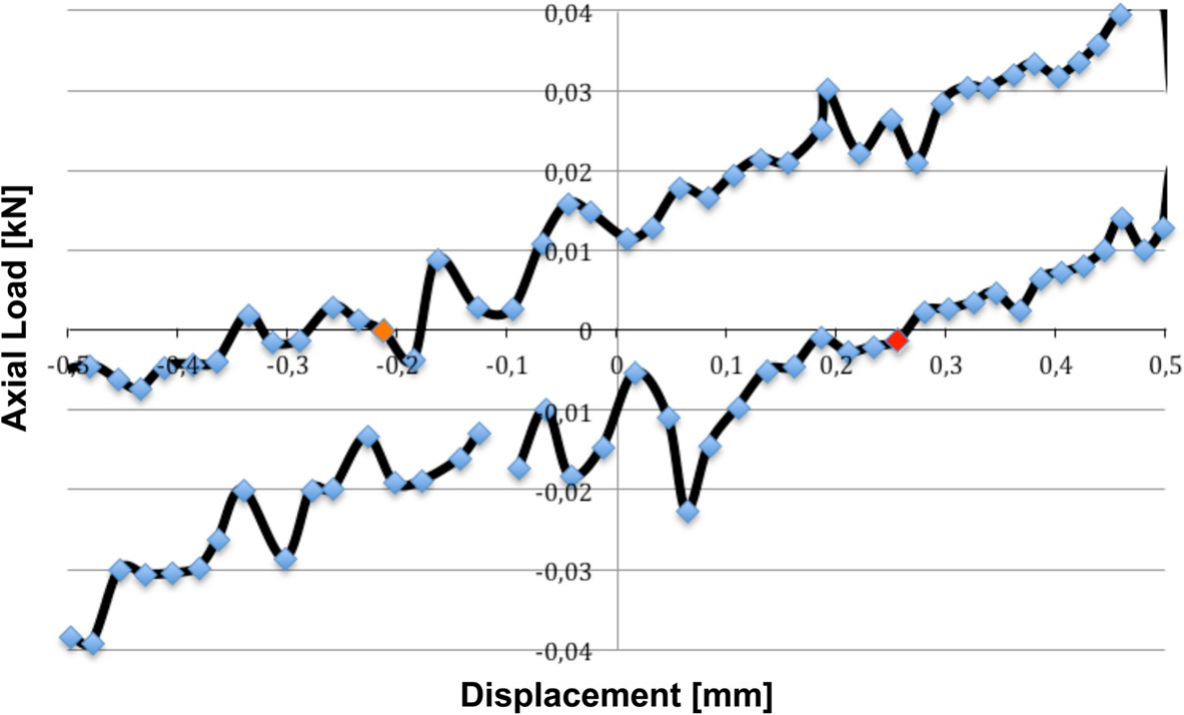
Load-Displacement curve of varus/valgus testing to determine neutral zone of an exemplary specimen. The *red* marked points were used for calculation of the neutral zone

The general setup motion can be easily described in terms of range of motion (ROM) and neutral zone (NZ) [18–21]. The neutral zone is defined as that region where motion is possible without any loading. Both parameters were determined for all quasi-static tests from the machine data plotting every singe cycle in Excel (Microsoft Corporation, Version 14, Redmond WA, USA) and measuring manually (Fig. 5). From the optical measurement system, the ROM was differentiated, and rotational motion was calculated between tibia and calcaneus (TIB-CAL), tibia and talus (TIB-TAL), talus and calcaneus (TAL-CAL). These results have mathematically the relation TIB-CAL = TIB-TAL + TAL-CAL. The results from the optical measurement system mathematically could not be transferred to the machine data, especially not for varus/valgus and flexion, due to the lever arm the extent of machine actuator movement is much higher then the movement in the bone-implant-construct.

Rotation about the x-axis represents varus/valgus movements, rotation about the y-axis dorsi-/plantarflexion, and rotation about the z-axis internal-/external rotation.

For statistical analysis ROM values from the Instron were compared to the Optotrak results (TIB-CAL) using the Wilcoxon signed-rank test for depended samples was used. The same procedure was used for statistical comparison within the Optotrak results.

## Results

### Torsion

**Fig. 6:**
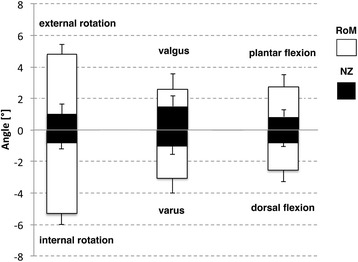
Range of motion and neutral zone values in degrees for the torsional, varus/valgus and flexion tests calculated from the material testing machine data as mean values with standard deviation

The mean range of motion calculated from the material testing machine data for internal and external rotation was 10.12° (SD 0.6). In comparison, the torsional range of motion as measured with the Optotrak system between tibia and calcaneus was 10° (SD 2.83). This difference was statistically not significant (*p* = 0.463). With the use of Optotrak, we were also able to determine the movements between tibia-talus and talus-calcaneus and found a 40 % significant higher rotation in the talocrural joint (*p* = 0.028; Table 1). In contrast, the machine data allowed us to calculate a neutral zone of 1.85° (Fig. 6).

**Table 1:** Summary of the results from all quasi-static tests

Torsion						
Instron machine				Optotrak® system		
ROM [°]		NZ [°]		TIB-CAL	TIB-TAL	TAL-CAL
10.12 (0.57)		1.85 (0.71)		10 (2.83)^b^	6.2 (1.82)^b^	3.8 (1.19)^b^
IR	ER	IR	ER	*p* = 0.028 *p* = 0.046		
5.32 (0.68)	4.8 (0.62)	0.84 (0.36)	1.0 (0.63)	*p* = 0.028		
Varus/valgus						
Instron machine				Optotrak® system		
ROM [°]		NZ [°]		TIB-CAL	TIB-TAL	TAL-CAL
5.65 (1.84)^a^		2.54 (0.99)		2.9 (0.46)^ab^	0.7 (0.07)^b^	2.2 (0.46)^b^
Varus	Valgus	Varus	Valgus	*p* = 0.028 *p* = 0.028		
3.05 (0.97)	2.6 (0.95)	1.04 (0.51)	1.5 (0.67)	*p* = 0.028		
Dorsal/Plantar flexion						
Instron machine				Optotrak® system		
ROM [°]		NZ [°]		TIB-CAL	TIB-TAL	TAL-CAL
5.30 (1.45)^a^		1.69 (0.58)		2.1 (0.39)^ab^	1.6 (0.27)^b^	0.5 (0.21)^b^
Dorsal Fl.	Dlantar Fl.	Dorsal Fl.	Plantar Fl.	*p* = 0.028 *p* = 0.028		
2.55 (0.73)	2.75 (0.74)	0.85 (0.22)	0.84 (0.45)	*p* = 0.028		

Showing mean values (±standard deviation) under peak load. Rotational movement between tibia and calcaneus (TIB-CAL), tibia and talus (TIB-TAL), talus and calcaneus (TAL-CAL). Significant differences between Instron and Optotrak values marked with ^a^. Significant differences within the Optotrak results are marked with ^b^

### Varus/valgus

**Fig. 7:**
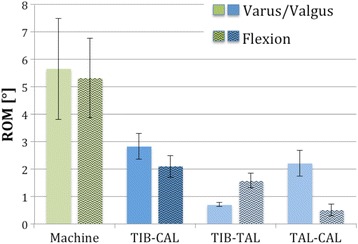
Range of motion in degrees for varus/valgus and flexion from machine data (*green*) and Optotrak (*blue*). TIB-CAL is the range of motion of the whole construct – measured between tibia and calcaneus. TIB-TAL is the movement in the upper ankle joint (tibia – talus) and TAL-CAL in the subtalar joint (talus – calcaneus)

The mean range of motion from the machine data observed here was 5.65° (SD 1.84) compared to 2.82° (SD 0.46) as measured using the Optotrak. This difference was statistically significant with *p* = 0.028. The subtalar joint showed a 70 % significant higher movement compared to the talocrural joint for varus/valgus bending (*p* = 0.028, Fig. 7).

### Dorsal/plantar flexion

The mean range of motion for the flexion test was 5.3° (SD 1.45) seen from the material testing machine and 2.1° (SD 0.39) as seen from the Optotrak. This difference was statistically significant with *p* = 0.046. We found approximately a 3-times significant higher movement in the talocrural joint compared to the subtalar joint (*p* = 0.028, Fig. 7).

For varus/valgus and flexion data, the material testing machine data enabled determination of values in the neutral zone (Fig.6).

## Discussion

TTCA using intramedullary nails is an established salvage procedure. Unfortunately, it happens quite often that non-union complications of the upper ankle joint and/or the subtalar joint occur.

Therefore, biomechanical testing and the development of new fixation methods and designs are of significant interest also for TTCA. Starting with femoral nails for TTCA, new generations of intramedullary nails have been developed again and again.

Biomechanical testing of the new developed implants was conducted in several test setups ranging from static to cyclic loading, but all of them mainly using machine data for mechanical characterization [3, 4, 22–25]. Fragomen et al. investigated the Ilizarov external fixator and an intramedullary nail regarding micro motions. Therefore they used a passive marker optical measurement system above and under the osteotomy gap, but only a two dimensional evaluation was possible [26].

With our modifications of the test setup developed by Mückley et al., we were able to exclude shear stress on the construct and ensure a constant lever arm and consequently a standardized application of constant moments/forces to the construct [10, 11]. The additional use of the 3D motion measurement system allows the detection of movements between the involved bones with high precision. Utilizing a combination of material testing machine data and the Optotrak system, it is possible to characterize the ROM from both, the neutral zone (taken from the machine data) and the interbony movements (taken from the Optotrak system). This is possible by matching the load data of the machine with the motion data of the Optotrak.

For the HAN we found the talocrural joint prone to torsional stress as well as dorsiflexion and plantarflexion, whereas the subtalar joint is more prone to varus/valgus stress. Comparing the range of motion results from the machine and the optical system, we found no significant differences for torsional loading. In contrast, we found statistically significant differences for varus/valgus and dorsal/plantar flexion. These differences result from the test setup with using a lever arm for these loading scenarios. Therefore, the values from the machine are significant higher compared with the values from the optical measurement system looking at pure bony movements. The results of the neutral zone are only determined from the machine data and therefore, include the instability of implant and of the fixture.

Further important features of today’s intramedullary hindfoot nails are locking and compression mechanisms. Biomechanical studies have demonstrated that a higher stability can be attained when a combination with these fixation methods is employed [10, 11].

Using the presented setup biomechanical investigations of the hindfoot can be performed very sophisticated. The optical measurement system allows the differentiation of the involved joints (tibio-talar and talo-calcanear) combined with the machine data, that allow gathering information of the neutral zone and the whole construct. Overall, the setup allows detecting weak points of implants or effects of operative procedures. The test setup is not limited to hindfoot arthrodesis and intramedullary nails. With slight modifications implants/operations concerning the ankle joint or the subtalar joint and the effect to the other joint can be investigated.

Using the results strategies/developments can be performed to optimize implants or procedures to improve the management of hindfoot pathologies. Moreover, modifications to the implants themselves may allow a further reduction of the complication rates by increasing the stability of the TTCA construct.

This study also has limitations: using biomechanical testing without any soft tissue/muscle/tendon simulation is a basic method to compare mechanical characteristics of bone implant interfaces and limited cofounders. Additionally, the cartilage in talocrural and the subtalar joint has not been removed. This allowed us a very standardized approach resulting in a relative unstable situation. Another limitation is the independence of the optical and machine data, this does us not allow to calculate neutral zone values from the optical dataset.

## Conclusion

The modified test setup for the hindfoot presented here promotes a more physiological and biomechanical loading. This offers – together with the 3D optical measuring system – a more standardized testing, above all for difficult cases, and also allows a detailed characterisation of the bone-implant constructs. Utilizing this, new implants and surgical techniques at the hindfoot can be compared with the current state of affairs; implant developments can be followed and, where necessary, their complications reduced.

## Abbreviations

HAN, hindfoot arthrodesis nail; NZ, neutral zone; ROM, range of motion; SD, standard deviation; TTCA, tibiotalocalcaneal arthrodesis
